# Probable Post Traumatic Stress Disorder in Kenya and Its Associated Risk Factors: A Cross-Sectional Household Survey

**DOI:** 10.3390/ijerph121013494

**Published:** 2015-10-26

**Authors:** Rachel Jenkins, Caleb Othieno, Raymond Omollo, Linnet Ongeri, Peter Sifuna, James Kingora Mboroki, David Kiima, Bernhards Ogutu

**Affiliations:** 1 Health Services and Population Research Department, Institute of Psychiatry, Kings College London, de Crespigny Park, London SE5 8AF, UK; 2 Department of Psychiatry, University of Nairobi, Kenya, Kenyatta National Hospital, Nairobi. P. O. Box 19676-00202; E-Mail: cjothieno@uonbi.ac.ke; 3 Kenya Medical Research Institute, P.O. Box 54-40100 Kisumu, Kenya; E-Mails: romollov@yahoo.co.uk (R.O.); longeri@kemri.org (L.O.); psifuna@yahoo.com (P.S.); ogutu6@gmail.com (B.O.); 4 Kenya Medical Training College, Nairobi P.O.Box 30195-00100, Kenya; E-Mail: jkmboroki@yahoo.com; 5 Ministry of Health, Nairobi P.O. Box 30016-00100, Kenya; E-Mail: dmkiima@gmail.com

**Keywords:** post-traumatic stress disorder, common mental disorder, Kenya, household survey, health and demographic surveillance systems

## Abstract

This study aimed to assess the prevalence of probable post-traumatic stress disorder (PTSD), and its associated risk factors in a general household population in Kenya. Data were drawn from a cross-sectional household survey of mental disorders and their associated risk factors. The participants received a structured epidemiological assessment of common mental disorders, and symptoms of PTSD, accompanied by additional sections on socio-demographic data, life events, social networks, social supports, disability/activities of daily living, quality of life, use of health services, and service use. The study found that 48% had experienced a severe trauma, and an overall prevalence rate of 10.6% of probable PTSD, defined as a score of six or more on the trauma screening questionnaire (TSQ). The conditional probability of PTSD was 0.26. Risk factors include being female, single, self-employed, having experienced recent life events, having a common mental disorder (CMD)and living in an institution before age 16. The study indicates that probable PTSD is prevalent in this rural area of Kenya. The findings are relevant for the training of front line health workers, their support and supervision, for health management information systems, and for mental health promotion in state boarding schools.

## 1. Background

Post-traumatic stress disorder is a relatively recent diagnostic construct and is characterised by flashbacks, nightmares, avoidance, numbing and hypervigilance [1]. It is different from other psychiatric disorders in that diagnosis requires that symptoms are caused by an external traumatic event. Traumatic events are distinct from and more severe than generally stressful life events. A traumatic event is where an individual experiences, witnesses or is confronted with life endangerment, death or serious injury to self, or close others. While some have questioned whether the construct of post-traumatic stress disorder (PTSD) is relevant for low income countries [2], in practice there are a number of studies which attest to its value [3]. Therefore the opportunity was taken to study the prevalence of symptoms of PTSD in Nyanza Province, in Kenya, as part of a wider study of mental disorders, immunity and malaria.

Kenya became a lower middle income country in 2014, when its GDP per capita rose from US $399 in 2000 to US $1040.55 in 2013, although poverty levels remain high at 45.9% [4]. Kenya’s population is growing rapidly by about 1 million a year from 10 million in 1969 to an estimated 43.2.million in 2013 (extrapolated from the 2009 census) [5], with nearly 50% aged under 15. Life expectancy at birth is 57 years, the adult literacy rate is 87%, the infant mortality rate is 55 per 1000 live births, the maternal mortality rate is 490 per 100,000 live births and HIV prevalence is currently estimated at 5.6% [6]. There is rapid urbanisation, the agricultural sector is highly inefficient, and the food supply is vulnerable to catastrophic drought and floods. Kenya has also suffered episodes of political turbulence, especially in Nyanza province in 2007 and again to a lesser extent in 2013.

Nyanza Province has relatively high levels of unplanned pregnancies (53%), deaths of children under five per 1000 live births (14.9%), spousal abuse (60%) [6,7], and considerably higher prevalence compared to the rest of Kenya of HIV of 17.7% in women and 14.1% in men [7,8], all of which socioeconomic and health challenges may impact on the mental health of the population.

The research was conducted as part of an overall collaborative programme of work between the Kenya Ministry of Health and the UK Institute of Psychiatry, Kings College London over the last 15 years, (including collaborations with the Kenya Medical Training College (KMTC), the Kenya Medical Research Institute (KEMRI) ,the Kenya Psychiatric Association, and Great Lakes University) comprising situation appraisal [9,10,11,12,13], studies of traditional healers [14], community health workers [15], district health workers [16], policy development [17], primary care training [18,19] and its evaluation [20,21,22,23]. This repeat epidemiological survey is important to assess the sustained mental health needs in the general adult population of Kenya, in a region of Kenya which experienced serious election violence in August 2007 [24].

We hypothesised that probable PTSD would be prevalent in this area of Kenya which has experienced repeated election violence, and that rates would be higher in women and in those with higher numbers of life events, as has been found by studies of PTSD in other countries.

## 2. Methods

The study relies on data drawn from a community study of the prevalence of mental disorders, and their risk factors in the general population in Nyanza province, near Lake Victoria in Kenya.

### 2.1. Study Population

The sample frame is a subdivision in an endemic area for malaria in Kenya, namely the Maseno area in Kisumu county, Nyanza Province in western Kenya, Maseno has a population of 70,805 [25]. Females constitute 53% of the population. The mean household size is four people per household with a population density of 374 people/km^2^. The population is largely young with a mean age of 23 years. The population 0–14 years constitutes 46%, ages 15–64 years constitute 49% and ages 65+ years constitute 5%. The population is primarily black African, and the languages spoken are Luo (which is the predominant ethnic group), Kiswahili and English. The area is largely rural, with most residents living in villages, which are a loose conglomeration of family compounds near a garden plot and grazing land. The majority of the houses are mud-walled with either grass thatched or corrugated iron-sheet roofs. Water is sourced mainly from community wells (40%), local streams (43%) and the lake (5%) for those mostly living on the shores of Lake Victoria [25]. Most water sources are not chlorinated. Subsistence farming, animal husbandry and fishing are the main economic activities in the area. Malaria is holoendemic in this area, and transmission occurs throughout the year. The “long rainy season” from late March to May produces intense transmission from April to August. The “short rainy season” from October to December produces another, somewhat less intense, transmission season from November to January.

### 2.2. Study Site

**Figure 1:**
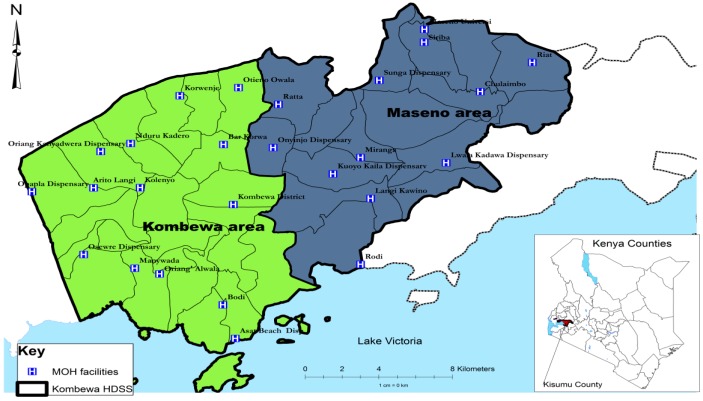
Location of the study site.

Figure 1 shows the study site, which is Maseno area, in Kisumu County, western Kenya

### 2.3. Study Participants

The study sample was selected from Maseno Area within Kisumu County, western Kenya. Maseno Area is sub-divided into four locations, 17 sub-locations and 184 enumeration areas (villages) based on mapping work done earlier by the Kombewa Health and Demographic Surveillance System (Kombewa HDSS) run by the Kenya Medical Research Institute (KEMRI)/Walter Reed Project (WRP). The Kombewa HDSS is a longitudinal population registration system set up to monitor the evolving health and demographic problems of the study population in Kombewa and Maseno areas [25]. Some villages with less than 50 households were merged together to create new enumeration areas, so that the final total of enumeration areas was 170. A random sample of seven households was drawn from each enumeration area, to give a projected sample of 1190 households, and hence 1190 adults. Village maps were used to assign households and guide the research assistants during the survey. Using the Kish Grid Method, one individual was selected at random from each of the sampled households [26]. Thus only one individual per household was interviewed. A total of 1190 households were visited, and a total sample of 1147 participants agreed to be interviewed. The demographics and reasons for the refusal were recorded in notebooks by the research assistants.

### 2.4. Study Procedures

Meetings were held with community leaders to explain the purpose of the survey, and answer questions. The heads of the sampled households, and then the identified participants in the survey were approached in their own homes for informed written and witnessed consent to the interview. The interview was administered by one of a group of 20 research assistants using a personal digital assistant (PDA), on which the interview questions were programmed in English, Kiswahili and Dholuo, and responses were recorded. The research assistants received a 5-day training course, and were supervised in the field by a field manager.

The participants received a structured epidemiological assessment of common mental disorders, and symptoms of PTSD , accompanied by additional sections on socio-demographic data, life events, social networks, social supports, disability/activities of daily living, quality of life, use of health services, and service use, adapted from the adult psychiatric morbidity schedule [27] used in the UK mental health survey programme. Demographic information collected included age, sex, ethnicity, marital status and household status (head, spouse or other). Socio-economic factors assessed included employment status, education attainment, economic assets and type of housing. All items were reviewed by Kenya colleagues to ensure content validity and applicability to the local context.

Respondents were asked whether or not a traumatic event had happened to them at any time in their life. To clarify the nature and severity of traumatic stressor that should be included, respondents were given a structured list as follows “The term traumatic event or experience means something like a major natural disaster, a serious automobile accident, being raped, seeing someone killed or seriously injured, having a loved one die by murder or suicide, or any other experience that either put you or someone close to you at risk of serious harm or death.” Those who had experienced a major trauma were asked when this had occurred. If after the age of 16, then the presence of post-traumatic stress disorder symptoms were assessed by the trauma screening questionnaire (TSQ) [28], a short screening tool designed to identify probable cases of current PTSD. The TSQ consists of the re-experiencing and arousal items from the Post-traumatic Stress Symptom Scale-Self report, which is aligned to DSM-IV criteria. It does not cover the DSM-IV criteria related to avoidance and numbing, but was selected as it has been found in previous studies to perform well as a predictor of clinical PTSD [29,30].

Common mental disorders were assessed by the Clinical Interview Schedule- Revised (CIS-R) [31], a gold standard instrument for use by lay interviewers to assess psychopathology in community settings. It has been widely used in high [32,33,34] and low income countries [35,36,37], including Tanzania [38,39] and Kenya [12]. The CIS-R measures the presence of 14 symptom-types in the preceding month and the frequency, duration and severity of each symptom in the past week. Scores, taken together with algorithms based on the ICD-10 [1], provide diagnoses of depressive episode (mild, moderate or severe), obsessive compulsive disorder, panic disorder, phobic disorder, generalised anxiety disorder and mixed anxiety/depressive disorder.

Respondents were given a list of 11 different stressful life events and asked to say which, if any, they had experienced in the last six months. The list included health risks (serious illness, injury or assault to self, or close relative), loss of a loved one (death of a relative; death of a close friend), relationship difficulties (separation or divorce; serious problem with a close friend or relative); income instability (being made redundant or sacked; having looked for work for over a month; loss of the equivalent of three months’ income) and legal problems (problems with the police involving a court experience; something of value lost or stolen). The list was developed for the British psychiatric morbidity survey programme [32,33,34], and slightly modified for the east African context. Scores were grouped into “none”, “one”, “two” and “three or more” life events and were also analysed by category.

Perceived lack of social support was assessed from respondents’ answers to seven questions which were used in the 1992 Health Survey for England [40], and the Office of National Statistics (ONS)Surveys of Psychiatric Morbidity [32,33]. The seven questions take the form of statements that individuals could say were not true, partly true or certainly true for them, in response to the question “There are people I know who”: (i) Do things to make me happy; (ii) Who make me feel loved; (iii) Who can be relied on no matter what happens; (iv) Who would see that I am taken care of if I needed to be; (v) Who accept me just as I am; (vi) Who make me feel an important part of their lives; and (vii) Who give me support and encouragement. Results were categorised into no, moderate or severe lack of perceived social support.

Social network size was assessed by respondents answers to three questions which have also been used in the ONS surveys of Psychiatric morbidity, namely: (i) How many adults who live with you do you feel close to; (ii) how many relatives aged 16 or over who do not live with you do you feel close to; (iii) how many friends or acquaintances who do not live with you would you describe as close or good friends. Responses were added into a total social network score.

Specific questions were also asked about caring responsibilities (Do you give care due to long term physical or mental disorder or disability? And if yes, amount of time spent giving care in a week); about growing up with one natural parent or two until age 16; and about spending time in an institution before the age of 16.

### 2.5. Statistical Analysis

We examined the prevalence of probable PTSD. The TSQ is scored by giving one point to each item experienced twice or more in the last week: a total of six or more out of the possible ten indicated a positive screen for PTSD. All respondents with a score of five or less were designated as screen negative [28]. An actual diagnosis of PTSD would require a full clinical assessment for PTSD. We calculated the conditional probability of screening positive for PTSD given that a severe trauma has previously occurred [41]. We also examined the predictors of probable PTSD, using STATA [42] to calculate unadjusted and adjusted odds ratios. A score of 12 or more across the 14 sections of the CIS-R was considered an indication of any common mental disoeder (CMD). [31,33,34]. Life event scores were grouped into none, one, two, and three or more life events.

Households have been categorized into different socio-economic levels using an index of household assets, constructed applying the principal component analysis procedure, as a proxy indicator for socio-economic status. In developing the asset quintiles, type of house, roofing & walling material, source of water, toilet facility and land have been used [43,44]. Household size was grouped into 1–6, and over six because almost half the population lives in households composed of at least seven members. Age was grouped into youth (16–30), middle age (30–60) and older age (60 plus) as these are meaningful age bands and our sample size was not large enough to give enough power if subdivided more extensively.

### 2.6. Ethics

Ethical approval was granted by the Kings College London (KCL) and KEMRI boards of research ethics respectively (PNM/11/12-54, SSC2374), and permission was obtained to conduct the study in households in Maseno area, which is part of the KEMRI/WRP in Kombewa HDSS. Written and witnessed informed consent was asked of participants to take part in the study.

## 3. Results

A total of 1190 households were selected, and 1158 participants consented to the study while 32 refused to participate in the study interviews, giving a response rate of 97.3%.

Table 1 shows the frequency of individual PTSD symptoms in the last week, the range of PTSD scores and the prevalence of probable PTSD using a cut off score of six or more.

Table 2 shows the relationship of probable PTSD (defined as score of six or more on the TSQ) with a range of sociodemographic, physical and psychosocial variables.

**Table 1:** Frequency of individual items on the trauma screening questionnaire (TSQ) and the prevalence of probable post-traumatic stress disorder (PTSD) (TSQ score 6+).

Trauma Screening Questionnaire		Scores		
Has a traumatic event or experience ever happened to you at any time in your life		Yes		552 (48.2)
		No		528 (46.1)
		Don’t apply		66 (5.8)
Have you experienced, at least twice in the past week: Feeling upset by reminders of the event		Yes		250 (50.1)
		No		241 (48.3)
		Don’t apply		8 (1.6)
Have you experienced, at least twice in the past week: Bodily reactions (such as fast heartbeat, stomach churning, sweatiness, dizziness) when reminded of the event		Yes		166 (33.3)
		No		317 (63.5)
		Don’t apply		16 (3.2)
Have you experienced, at least twice in the past week: Difficulty falling or staying asleep		Yes		191 (38.3)
		No		297 (59.5)
		Don’t apply		11 (2.2)
Have you experienced, at least twice in the past week: Irritability or outbursts of anger		Yes		155 (31.2)
		No		334 (67.2)
		Don’t apply		8 (1.6)
Have you experienced, at least twice in the past week: Difficulty concentrating		Yes		144 (29.0)
		No		344 (69.2)
		Don’t apply		9 (1.8)
Have you experienced, at least twice in the past week: Heightened awareness of potential dangers to yourself and others		Yes		117 (23.6)
		No		360 (72.6)
		Don’t apply		19 (3.8)
Have you experienced, at least twice in the past week: Being jumpy or being startled at something unexpected		Yes		102 (20.5)
		No		378 (75.9)
		Don’t apply		18 (3.6)

Total PTSD scores	0 to 2		803 (70.1)	
	3 to 5		222 (19.4)	
	6 to 7		56 (4.9)	
	Above 7		65 (5.6)	
Prevalence of probable PTSD (TSQ 6+): **% (95% CI)**			10.6 (8.8–12.5)	

The prevalence of probable PTSD, defined as a score of six or more on the TSQ, was 10.6%. The conditional probability of PTSD was 26%.

Risk factors significant in the bivariate analysis shown in Table 2 included being female (OR 2.4, *p* < 0.001), being widowed or divorced (OR 1.6, *p* = 0.041), self-employed (OR 1.8, *p* = 0.005), experiencing life events (OR 2.1, *p* = 0.008 for 2–3 life events, and OR 4.1, *p* < 0.001 for four or more life events ), having any CMD (OR 8.0, *p* < 0.001), and spending time in an institution before the age of 16 (OR 2.0, *p* = 0.001).

**Table 2:** Prevalence of probable PTSD over the last one week and its relationship with socio-demographic and health related factors, using univariate analysis (unadjusted odds ratios).

Factors		N	Prevalence: *n* (%)	Unadjusted OR (95% C.I)	*p*-Value
Prevalence of PTSD		1146	121 (10.6)		
Sex	Male	601	40 (6.7)	1	-
	Female	545	81 (14.9)	2.4 (1.64–3.65)	<0.001
Age group	<30 years	281	39 (13.9)	1	-
	30–60 years	448	52 (11.6)	0.8 (0.52–1.27)	0.367
	>60 years	171	23 (13.5)	1.0 (0.55–1.68)	0.898
Household size	≤6 people	566	61 (10.8)	1	-
	>6 people	580	60 (10.3)	1.0 (0.66–1.39)	0.812
Marital Status	Married/cohabiting	714	65 (9.1)	1	-
	Single	183	22 (12.0)	1.4 (0.82–2.28)	0.236
	Widowed/divorced	248	34 (13.7)	1.6 (1.01–2.47)	0.041
Education	None	131	15 (11.5)	1	-
	Primary	625	68 (10.9)	0.9 (0.52–1.71)	0.849
	Secondary	319	35 (11.0)	1.0 (0.50–1.81)	0.883
	Post secondary	71	3 (4.2)	0.3 (0.10–1.22)	0.098
Employment status	Unemployed	563	47 (8.4)	1	-
	Self employed	484	67 (13.8)	1.8 (1.19–2.62)	0.005
	Employed	99	7 (7.1)	0.8 (0.37–1.91)	0.669
	Positive	269	30 (11.2)	1.1 (0.72–1.79)	0.584
Asset Groups	Lowest, Q1	403	35 (8.7)	1	-
	Q2	409	50 (12.2)	1.5 (0.93–2.31)	0.101
	Highest, Q3	334	36 (10.8)	1.3 (0.78–2.07)	0.338
Life events	0–1	361	18 (5.0)	1	-
	2–3	476	48 (10.1)	2.1 (1.22–3.74)	0.008
	4 or more	309	55 (17.8)	4.1 (2.37–7.20)	<0.001
Perceived lack of social support	No lack: 0	3	1 (33.3)	1	-
	Moderate lack: 1–7	312	31 (9.9)	0.2 (0.02–2.50)	0.223
	Severe lack: 8+	828	89 (10.8)	0.2 (0.02–2.68)	0.247
Total social group size	3 or less	144	12 (8.3)	1	-
	–8	519	56 (10.8)	1.3 (0.69–2.56)	0.391
	9 or more	480	53 (11.0)	1.4 (0.71–2.63)	0.352
Presence of CMD	No	1027	75 (7.3)	1	-
	Yes	119	46 (38.7)	8.0 (5.16–12.39)	<0.001
Carer for >4 h	No	26	3 (11.5)	1	-
	Yes	171	14 (8.2)	0.7 (0.18–2.56)	0.573
Spent time at institution <16 years	No	919	84 (9.1)	1	-
	Yes	220	37 (16.8)	2.0 (1.32–3.05)	0.001
Did not live continuously with both natural parents until age 16	No	963	102 (10.6)	1	-
	Yes	176	19 (10.8)	1.0 (0.61–1.72)	0.936

Risk factors remaining significant in the adjusted analysis shown in Table 3 include being female (OR 2.0, *p* = 0.004), single (OR 1.8, *p* = 0.038). self-employed (OR 1.9, *p* = 0.003), having experienced recent life events (OR 2.1, *p* = 0.013 for 2–3 life events, and OR 3.8, *p* < 0.001 for four or more life events), having a CMD (OR 7.0, *p* < 0.001) and living in an institution before age 16 (OR 1.8, *p* = 0.018).We also examined the risk of probable PTSD following individual life events (see Table 4) and found that those life events significantly associated with probable PTSD were serious illness, injury or assault to self (OR 1.8, *p* = 0.03); to a close relative (OR 2.4, *p* < 0.001); death of an immediate family member (OR 2.1, *p* = 0.001)). Separation due to marital differences, divorce or steady relationship broken (OR 2.6, *p* = 0.033); major financial crisis, like losing an equivalent of three months’ income (OR 1.6, *p* = 0.023); something you valued being lost or stolen (OR 3.5, *p* < 0.001); Violence at home (OR 3.0, *p* < 0.001); and running away from home (OR 4.5, *p* < 0.001).

**Table 3:** The final adjusted model risk factors for PTSD, using multivariate logistic regression analysis (adjusted odds ratios).

Factors		Adjusted OR * (95% C.I)	*p*-Value
Gender	Female	2.0 (1.25 to 3.17)	0.004
Marital status	Single	1.8 (1.03 to 3.13)	0.038
	Widowed/divorced	0.7 (0.44 to 1.29)	0.294
Employment	Self employed	1.9 (1.24 to 3.00)	0.003
	Employed	1.1 (0.47 to 2.77)	0.770
Total life events	2–3	2.1 (1.17 to 3.87)	0.013
	4 or more	3.8 (2.10 to 6.89)	<0.001
Any CMD		7.0 (4.21 to 11.68)	<0.001
Spent time at institution <16 years		1.8 (1.10 to 2.79)	0.018

**Table 4:** Relationship of probable PTSD with individual life events, using univariate analysis.

Factors		N	Prevalence: *n* (%)	Unadjusted OR (95% C.I)	*p*-Value
Serious illness, injury or assault to self	No	818	72 (8.8)	1	-
	Yes	328	49 (14.9)	1.8 (1.23 to 2.68)	0.003
Serious illness, injury or assault to a close relative	No	819	65 (7.9)	1	-
	Yes	327	56 (17.1)	2.4 (1.63 to 3.52)	<0.001
Death of an immediate family member of yours	No	443	29 (6.6)	1	-
	Yes	703	92 (13.1)	2.1 (1.39 to 3.32)	0.001
Death of a close family friend or other relative	No	693	75 (10.8)	1	-
	Yes	453	46 (10.2)	0.9 (0.63 to 1.37)	0.719
Separation due to marital differences, divorce or steady relationship broken	No	1115	114 (10.2)	1	-
	Yes	31	7 (22.6)	2.6 (1.08 to 6.08)	0.033
Serious problem with a close friend, neighbour or relative	No	1046	109 (10.4)	1	-
	Yes	100	12 (12.0)	1.2 (0.62 to 2.21)	0.624
Being made redundant or sacked from your job	No	1093	119 (10.9)	1	-
	Yes	53	2 (3.8)	0.3 (0.08 to 1.34)	0.118
Looking for work without success for >1 month	No	997	106 (10.6)	1	-
	Yes	149	15 (10.1)	0.9 (0.53 to 1.66)	0.834
Major financial crisis, like losing an equivalent of 3months income	No	881	83 (9.4)	1	-
	Yes	265	38 (14.3)	1.6 (1.07 to 2.43)	0.023
Problem with police involving court appearance	No	1108	114 (10.3)	1	-
	Yes	38	7 (18.4)	2.0 (0.85 to 4.57)	0.115
Something you valued being lost or stolen	No	990	83 (8.4)	1	-
	Yes	156	38 (24.4)	3.5 (2.29 to 5.41)	<0.001
Bullying	No	1093	113 (10.3)	1	-
	Yes	53	8 (15.1)	1.5 (0.71 to 3.35)	0.275
Violence at work	No	1098	117 (10.7)	1	-
	Yes	48	4 (8.3)	0.8 (0.27 to 2.16)	0.609
Violence at home	No	932	76 (8.2)	1	-
	Yes	214	45 (21.0)	3.0 (2.00 to 4.49)	<0.001
Sexual abuse	No	1140	121 (10.6)	1	-
	Yes	6	0 (-)	-	-
Being expelled from school	No	1113	116 (10.4)	1	-
	Yes	33	5 (15.2)	1.5 (0.58 to 4.05)	0.387
Running away from you home	No	1119	112 (10.0)	1	-
	Yes	27	9 (33.3)	4.5 (1.97 to 10.24)	<0.001
Being homeless	No	1132	118 (10.4)	1	-
	Yes	14	3 (21.4)	2.3 (0.64 to 8.52)	0.196

## 4. Discussion

### 4.1. Overall Findings

The study found an overall prevalence rate of 10.6% of PTSD, defined as a score of six or more on the TSQ. The conditional probability of PTSD was 26%. Risk factors remaining significant in the adjusted analysis include being female, single, self-employed, having experienced recent life events, having a CMD and living in an institution before age 16. The key traumatic life events associated with PTSD were serious illness, injury or assault to self; or to a close relative; death of an immediate family member; separation due to marital differences, divorce or steady relationship broken; major financial crisis, like losing an equivalent of three months’ income; something valued being lost or stolen; violence at home; and running away from home.

### 4.2. Comparison with Other Relevant Studies

#### Prevalence 

Comparisons of prevalence rates between studies are hampered by use of different instruments, symptom time periods, and samples. Our finding of a one week prevalence of PTSD 10.6% is lower than the rate of 34.5% one year prevalence (30.5% in boys and 42.3% in girls) aged from 13 to 20 years old, found in a study of Kenyan pupils in secondary boarding schools [45], the rate of 50.5% found in Kenya high school students aged 12–26 [46] and the rate of 65.7% found in Mau Mau concentration camp survivors [47].

In the African studies we have identified, rates were very variable, with higher rates in conflict and post-conflict areas. A household study in Rwanda, eight years after the civil war, found a rate of 24.8% past month prevalence of PTSD [48] and a study in the Nile delta of a refugee war population found prevalence rates of 31.6 and 40.1 in males and females respectively [49], whereas a South African national mental health survey found much lower rates of 2.3% life time and 0.7% one year prevalence [50]. However, a study of South African urban secondary schools found a rate of 22% [51].

There are several methodological reasons why our prevalence of probable PTSD may be artefactually low. PTSD symptoms were only assessed when a potentially traumatic event had occurred after the age of 16, but childhood exposure to trauma may have occurred for some individuals who did not acknowledge trauma after the age of 16. Childhood trauma is strongly associated with adult PTSD, and therefore the estimate of life time exposure to trauma and probable PTSD may be greatly underestimated. This possibility is reinforced by the high odds ratio of probable PTSD amongst individuals who reported having run away from home, which may be a proxy for childhood trauma. Secondly, serious injury or illness to self or others was treated as a life event and not as a potentially traumatic event. Given the strong odds ratios of Probable PTSD for both of these stressors, it is likely that many respondents who had experienced these events were incorrectly ruled out as not being probable PTSD cases as a result of them not being asked to complete the TSQ. Thirdly, the cardinal PTSD symptoms of intrusion and avoidance were endorsed by 33.5%, as were sleep disturbances (38%), so it appears that a substantial subgroup may have been classified as not having PTSD due to not endorsing hypervigilance, startle or concentration problems even though they were experiencing core PTSD symptoms. The adversities with which this population lives may lead to chronic vigilance or demands for rapid coping or re-direction of attention that in other circumstances would be considered abnormal but in this population may be accepted as normal. Finally, using only the last week as the time frame for symptoms may lead to substantial underestimate for current probable PTSD, and life time estimates would be several times higher. Had the estimated prevalence rate been based on all the cardinal PTSD symptoms, on past month symptoms and on lifetime symptoms, higher prevalence rates would have been found which would have been more comparable with the 20%–30% estimates found in other African studies in populations experiencing high adversity and conflict.

It is of interest to compare these relatively high prevalence rates in Africa with the much lower rates found in adult surveys in the UK and the US. In a national survey in the UK, a third (33.3%) of people reported having experienced a traumatic event in the UK since the age of 16. Experience of trauma in adulthood was higher in men (35.2%) than women (31.5%). Overall, 3.0% of adults screened positive for current PTSD. While men were more likely than women to have experienced a trauma; there was no significant difference by sex in rates of screening positive for current PTSD (2.6% of men, 3.3% of women) [40]. Similarly, a national survey in the US, the National Comorbidity Survey Replication (NCS-R), found 6.8% the lifetime prevalence and 3.5% current past year PTSD prevalence [52]. However it is noteworthy that the American survey did find a significant gender difference. Thus the lifetime prevalence of PTSD among men was 3.6% and among women was 9.7%. The twelve month prevalence was 1.8% among men and 5.2% among women [52].

We confirmed our hypotheses of a higher rate of probable PTSD in women, and a close relationship of probable PTSD with numbers of life events. The close association of probable PTSD with CMD has relevance for clinical assessment and treatment. The vulnerability conferred by living in an institution before the age of 16 is also worth noting. The associations with injury, violence and death are unsurprising and have been found in the world mental health survey [53]. Financial crisis and losing something valuable are likely to be extremely stressful in such a poor environment with few assets to cushion such problems, and an association with being robbed or mugged was reported in an urban survey in South Africa and Nairobi [51], and with destruction or loss of property in Rwanda [48].

### 4.3. Strengths of Study

The strengths of the study are the use of a health and demographic surveillance site for the random sample of households, the high response rate, and the systematic approach to the clinical and sociodemographic assessments. The population in the surveillance site is regularly monitored by field staff who visit each household bi-annually to capture health and demographic information (Birth rates, Death rates, Causes of Death, Pregnancies, Immunization status, in-and out-migrations, *etc*.). Various studies nested on the DSS platform take advantage of the sampling frame inherent in the HDSS, whether at individual, household/compound or regional levels. This familiarity with survey procedures is likely to have been influential in the achievement of a high response rate.

### 4.4. Limitations of Study

As always, the potential for measurement error when using screening instruments should be acknowledged, given self-reported experiences may be subject to recall or social desirability or cultural response bias [54]. The implementation of the study was hampered by a number of logistical challenges which included the difficult terrain, posing problems for local transport for research staff, and continuing administrative difficulties, which led to delays in the implementation of the project. The interviewing period, initially planned to last three months, took place over a period of six months, and was temporarily halted for several weeks over the period of the 2013 election due to further fears of election unrest.

## 5. Conclusions

The prevalence of probable PTSD in the general household adult population in this poor rural area of Kenya which has been subject to repeated election violence, is 10.6%, higher in women, those who are single, the self-employed, those with higher numbers of life events, those with CMD and those who spent time in an institution before age 16. These findings have implications for training of front line health workers, their support and supervision, for health management information systems, and for planning of services, including mental health promotion in state boarding schools.
